# Insulin-like growth factor-binding protein 7 (IGFBP7): A microenvironment-dependent regulator of angiogenesis and vascular remodeling

**DOI:** 10.3389/fcell.2024.1421438

**Published:** 2024-07-09

**Authors:** Kwok Keung Lit, Zhamilya Zhirenova, Anna Blocki

**Affiliations:** ^1^ Institute for Tissue Engineering and Regenerative Medicine, The Chinese University of Hong Kong, Shatin, Hong Kong SAR, China; ^2^ School of Biomedical Sciences, Faculty of Medicine, The Chinese University of Hong Kong, Shatin, Hong Kong SAR, China; ^3^ Center for Neuromusculoskeletal Restorative Medicine (CNRM), Hong Kong Science Park, Shatin, Hong Kong SAR, China; ^4^ Department of Orthopaedics and Traumatology, Faculty of Medicine, The Chinese University of Hong Kong, Shatin, Hong Kong SAR, China

**Keywords:** insulin-like growth factor-binding protein 7 (IGFBP7), luminogenesis, microenvironment, tissue remodeling, angiogenesis, vascularization

## Abstract

Insulin-like Growth Factor-Binding Protein 7 (IGFBP7) is an extracellular matrix (ECM) glycoprotein, highly enriched in activated vasculature during development, physiological and pathological tissue remodeling. Despite decades of research, its role in tissue (re-)vascularization is highly ambiguous, exhibiting pro- and anti-angiogenic properties in different tissue remodeling states. IGFBP7 has multiple binding partners, including structural ECM components, cytokines, chemokines, as well as several receptors. Based on current evidence, it is suggested that IGFBP7’s bioactivity is strongly dependent on the microenvironment it is embedded in. Current studies indicate that during physiological angiogenesis, IGFBP7 promotes endothelial cell attachment, luminogenesis, vessel stabilization and maturation. Its effects on other stages of angiogenesis and vessel function remain to be determined. IGFBP7 also modulates the pro-angiogenic properties of other signaling factors, such as VEGF-A and IGF, and potentially acts as a growth factor reservoir, while its actual effects on the factors’ signaling may depend on the environment IGFBP7 is embedded in. Besides (re-)vascularization, IGFBP7 clearly promotes progenitor and stem cell commitment and may exhibit anti-inflammatory and anti-fibrotic properties. Nonetheless, its role in inflammation, immunomodulation, fibrosis and cellular senescence is again likely to be context-dependent. Future studies are required to shed more light on the intricate functioning of IGFBP7.

## Introduction

The gene for insulin-like growth factor-binding protein 7 (IGFBP7) was initially identified and characterized as a gene suppressed in meningiomas. Today, IGFBP7 is also known under various aliases, including insulin-like growth factor binding protein-related protein 1 (IGFBP-rp1), MAC25, angiomodulin (AGM), tumor-derived adhesion factor (TAF), or prostacyclin-stimulating factor (PSF) (Murphy et al., 1993).

This 30 kDa secretory glycoprotein was classified as a member of the insulin-like growth factor-binding protein (IGFBP) superfamily, due to its ability to bind insulin growth factor (IGF). Noteworthily, it only shares about 20%–25% sequence homology with other proteins of the IGFBP family, while its ability to bind IGF was reported to be 100 times weaker than that of other IGFBPs (Oh et al., 1996).

IGFBP7 is expressed in various tissues and by various cell types such as peripheral nerves, smooth muscle cells, and epithelial cells, while lymphocytes, plasma cells, and adipocytes did not express IGFBP7. It was also noted that the intensity of IGFBP7 expression within the same cell type, such as kidney epithelial cells, varied spatially within the organ, suggesting that IGFBP7 may have specific functions in these organs (Akiel et al., 2014).

IGFBP7 is upregulated in the angiogenic vasculature during physiological (Li Y. et al., 2023) and some pathological processes, is expressed by endothelial cells, perivascular and stromal cells, and is predominantly localized in the extracellular matrix (ECM) (Akaogi et al., 1996a; Usui et al., 2002). There it binds to various ECM components such as collagen IV (Col IV) (Akaogi et al., 1996a) and heparan sulfate (Sato et al., 1999) and is well embedded within the vascular basement membrane (Akiel et al., 2014). It was also found to be stored in the Weibel Palade bodies of endothelial cells (Van Breevoort et al., 2012). IGFBP7 can bind to insulin (Oh et al., 1996), activin (Kato, 2000), various chemokines such as C-C motif chemokine 5 (CCL5), C-C motif chemokine 21 (CCL21), and C-X-C motif chemokine 10 (CXCL10) (Nagakubo et al., 2003), as well as growth factors such as vascular endothelial growth factor A (VEGF-A) (Usui et al., 2002) and IGF (Oh et al., 1996). Known receptors of IGFBP7 are IGF-I receptor (Evdokimova et al., 2012), integrin αvβ3 (Komiya et al., 2014), and CD93 (Sun et al., 2021), a C-type lectin transmembrane receptor.

IGFBP7 is mainly studied in tumorigenesis (Kato, 2000; Jiang et al., 2008; Chen R. Y. et al., 2010; Chen et al., 2015; Tomimaru et al., 2012; Rupp et al., 2015; Li et al., 2019; Li et al., 2023a; Zhang et al., 2019; Hong et al., 2023), acute kidney failure (Titeca-Beauport et al., 2019; Esmeijer et al., 2021; Yu et al., 2022), as well as in liver (Yan et al., 2019) and cardiovascular diseases (Zhang et al., 2022). Despite all efforts invested into researching IGFBP7, the biological function of this protein is not well understood. Within the tumorigenesis context, accumulating studies clarified that IGFBP7 is upregulated in some types of cancers, while downregulated in others (Liu Y. et al., 2023). These entirely different manifestations suggest that IGFBP7 may function as a “double-edged sword” in cancer cell growth and progression, displaying an ambiguous action in distinct types of cancers (Jin et al., 2020). For example, IGFBP7 was previously reported to act as an oncogene in some cancers (Jiang et al., 2008; Rupp et al., 2015; Li D. et al., 2023; Hong et al., 2023), but also exhibited tumor-suppressing properties in others (Kato, 2000; Chen R. Y. et al., 2010; Chen et al., 2015; Tomimaru et al., 2012; Li et al., 2019; Zhang et al., 2019). Likewise, the role of IGFBP7 in neovascularization has been the subject of significant research, and conflicting biological functions of IGFBP7 during angiogenesis have been described.

This review provides a new and critical perspective on the complex role of IGFBP7 in angiogenesis and vascular remodeling in embryonic development, as well as re-vascularization during physiological and pathological conditions. It also highlights new insights into the role of IGFBP7 in other tissue remodeling scenarios such as progenitor cell commitment, inflammation, immunomodulation, fibrosis and cellular senescence. Based on the critical analysis of current research studies, we conclude that IGFBP7 acts in unison with its ECM microenvironment, resulting in its biological activity being context-dependent.

### IGFBP7 in physiological vasculature formation

Upregulated IGFBP7 expression has been observed in activated vasculature during various biological processes including tissue injury and repair, wound healing, and during embryonic development. Here the angiogenic role of IGFBP7 will be dissected based on various remodeling vasculature scenarios, experimental approaches and key findings.

### IGFBP7 in vascular development

In humans, mutation of IGFBP7 was linked to familial retinal arterial macro aneurysms (Abu-Safieh et al., 2011). In an IGFBP7^lacZ/+^ mouse model IGFBP7 was expressed in the vasculature by E10.5 and increased in intensity throughout embryogenesis, suggesting that it may play a role in embryonic angiogenesis (Hooper et al., 2009). In a zebrafish model, whole mount *in situ* hybridization was utilized to examine the expression of IGFBP7 during zebrafish embryogenesis. IGFBP7 transcripts were detected in the vasculature of the head, eye, and trunk and more specifically in the vascular endothelium, although it was not exclusive to developing vasculature. Knockdown of IGFBP7 using morpholinos resulted in a multifactorial phenotype consistent with angiogenic defects, including an obvious reduction in blood flow through intersomitic vessels, which usually form *via* sprouting angiogenesis from the dorsal aorta. These defects in circulation were accompanied by pericardial oedema, suggestive of vascular sprouting and patterning defects (Hooper et al., 2009). Intriguingly, the IGFBP7 loss-of-function phenotype was strikingly similar to the phenotype of VEGF-A knockdown and their double knockdown resulted in synergistic effects, including strongly disrupted endothelial sprouting. When VEGF-A was overexpressed, the compensatory induction of the VEGF receptor 2 (VEGFR-2) was blocked by the simultaneous IGFBP7 knockdown, suggesting an interaction between IGFBP7 and VEGF-A, where IGFBP7 potentially modulates vascular remodeling in part by temporizing the pro-angiogenic effects of VEGF-A (Hooper et al., 2009).

A comparative transcriptome study that investigated the role of IGFBP7 in central nervous system (CNS) and blood-brain-barrier (BBB) development in mice at E13.5 revealed that IGFBP7 expression was significantly increased in cortical endothelial cells as compared to lung endothelial cells (Bar et al., 2020). Moreover, transcript levels of IGFBP7 in cortex endothelial cells were comparable to levels of the most commonly used BBB markers, while pan-endothelial markers exhibited similar levels in both endothelial cell types. Histological evaluation of protein levels confirmed that IGFBP7 was found in cortical vasculature, where it co-localized with endothelial cells and pericytes. Interestingly, IGFBP7 could still be detected in adult CNS vasculature, as well as CNS parenchyma. Indeed, researchers found that from embryonic stages E14.5 onwards IGFBP7 was expressed in all CNS vasculature, including choroid plexus vasculature, and therefore should not be considered as a BBB-specific marker or inducer at these stages.

**FIGURE 1:**
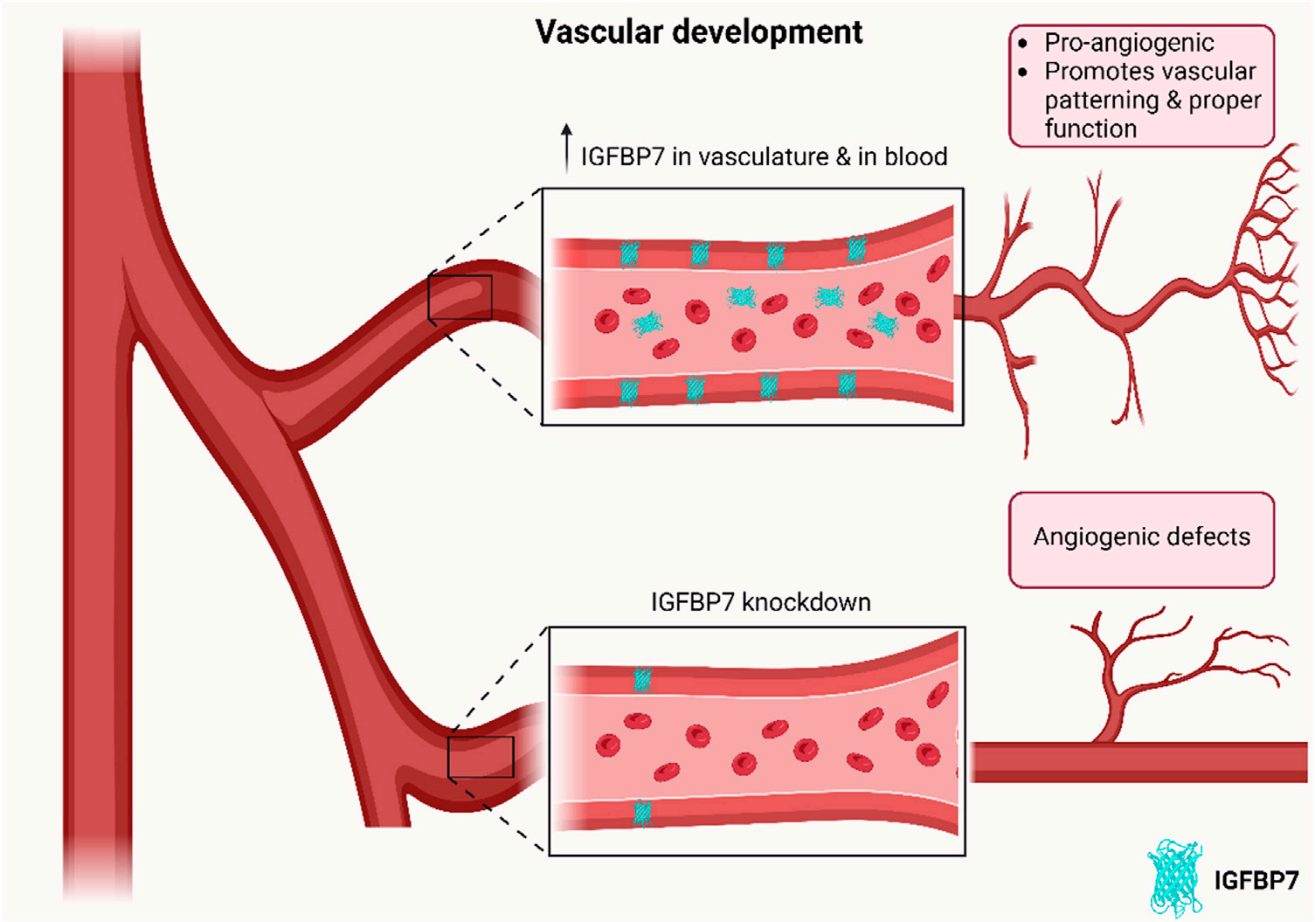
Schematic of effects of IGFBP7 on developing vasculature. IGFBP7 is upregulated in developing vasculature across organs and species, while its depletion results in angiogenic defects, suggesting that IGFBP7 may have a crucial role in related angiogenic processes in developing vasculature (Schematic was generated using BioRender.com with an academic license).

The consistent expression of IGFBP7 in developing vasculature across organs and species (Hooper et al., 2009; Bar et al., 2020), suggests that IGFBP7 may have a crucial role in related angiogenic processes. Especially since its knockdown resulted in reduced vascularization, oedema, and patterning defects (Hooper et al., 2009), it may be crucial for the formation of functional vasculature during development (Figure 1; Table 1).

**TABLE 1:** Effects of IGFBP7 on vascular development.

Administration/Experimental approach	Results	Potential effects on vascular development	References
Endogenous expression	• IGFBP7 mutation in human patients with autosomal recessive Familial Retinal Arterial Macroaneurysms (FRAM)	Potentially promotes vascular patterning and proper function	Abu-Safieh et al. (2011)
Endogenous expression	• Enhanced expression in the vasculature of E10.5 mouse embryo • Expression detected in the vasculature of the head, eye, and trunk in the zebrafish embryo • Global knockdown resulted in angiogenic defects in zebrafish embryo	Pro-angiogenic	Hooper et al. (2009)
Endogenous expression	• Expression detected in CNS vasculature of E12.5 embryo • Higher expression levels detected in cortex endothelial cells as compared to lung endothelial cells in E13.5 embryo	Pro-angiogenic	Bar et al. (2020)

### IGFBP7 in activated physiological post-natal vasculature

In IGFBP7^lacZ/+^ mice, IGFBP7 was upregulated in the granulation tissue of regenerating skin post-wounding and in neoangiogenic tufts in an oxygen-induced retinopathy model. In a model of hemangiogenesis, where myelosuppression causes an angiogenic response in bone marrow sinusoids, IGFBP7 was upregulated in sinusoidal endothelial cells (Hooper et al., 2009). Immunofluorescent staining also confirmed that IGFBP7 was upregulated in the vasculature of patients with post-traumatic brain injury (Wang et al., 2021) and stroke (Buga et al., 2014). These findings suggest that IGFBP7 was upregulated during physiological angiogenesis and may be involved in vascular repair and remodeling.

Co-injection of VEGF-A and soluble IGFBP7 sub-dermally (Miles assay) confirmed that VEGF-A-induced hyperpermeability of blood vessels could be abolished by IGFBP7, suggesting it may modulate VEGF-A mediated processes (Bar et al., 2020). Indeed, some studies exist that even reported anti-angiogenic properties of IGFBP7. *In vivo*, intraocular injection of soluble IGFBP7 in young post-natal mice resulted in a marked inhibition of retinal angiogenesis and severe patterning defects in retinal vessels, while early injection of soluble IGFBP7 into a calvarial bone defect in adult rats resulted in impaired angiogenesis and reduced bone mineral density (Sun et al., 2022).

*In vitro*, recombinant IGFBP7 inhibited VEGF-A- and luteinizing hormone (LH)-induced proliferation, migration, and tube formation of rat luteal microvascular endothelial cells (LECs), while IGFBP7 had no effects on any of the biological processes by itself (Tamura et al., 2009; Tamura et al., 2014). Another study demonstrated that treatment of human umbilical vein endothelial cells (HUVECs) with exogenous IGFBP7 could reduce their tube formation ability (Liu F. et al., 2023), spheroid sprouting, and downregulate CD34, VEGFR-2, C-X-C chemokine receptor type 4 (CXCR-4), and Ephrin B2 (EFNB2) expression (Sun et al., 2022). IGFBP7 was also reported to attenuate phosphorylation of mitogen-activated protein kinase (MAPK), extracellular signal-regulated kinase 1/2 (ERK1/2), VEGF-A-enhanced cyclooxygenase-2 (COX-2) mRNA expression and prostaglandin E2 (PGE2) secretion in HUVECs (Tamura et al., 2009) and rat LECs (Tamura et al., 2014; Tamura et al., 2014; Tamura et al., 2014; Tamura et al., 2014). Moreover, the knockdown of endogenous IGFBP7 in HUVECs enhanced COX-2 and VEGF mRNA expression (Tamura et al., 2009). Congruently, another study showed that the knockdown of IGFBP7 in rhesus monkey RF/6A choroidal endothelial cells promoted cell viability, cell migration and tube formation *in vitro*. This coincided with increased levels of VEGF and phosphorylated RAF/MAPK/ERK and the observed effect could be attenuated with the addition of exogenous IGFBP7 (Zhu et al., 2020).

Nonetheless, it is important to note that a range of studies exists that reported contrasting and pro-angiogenic effects of IGFBP7. As such, VEGF-A was reported to be able to induce IGFBP7 expression in HUVECs (Komiya et al., 2014). The knockdown of IGFBP7 gene expression inhibited tube formation in HUVECs, while the addition of exogenous IGFBP7 protein increased their tube formation and migration (Sun et al., 2021). Upon knockdown of IGFBP7 receptor CD93 in HUVECs, IGFBP7 protein lost this promoting effect (Sun et al., 2021). Similarly, neutralizing antibodies against IGFBP7 inhibited bovine capillary endothelial cell sprouting *in vitro* (Van Beijnum et al., 2006), while exogenous IGFBP7 addition promoted tube formation of human brain endothelial cells on Matrigel (Pen et al., 2008). This is in stark contrast to the above-mentioned studies (Tamura et al., 2009; Tamura et al., 2014).

Besides endothelial cells, various stromal cells including fibroblasts (Newman et al., 2011; Velázquez-Enríquez et al., 2021; Moita et al., 2022) and mesenchymal stem cells (MSCs) (Lam et al., 2022) are known to secrete IGFBP7. When HUVECs were co-cultured with adipose-derived stem cells (ASCs), optionally overexpressing IGFBP7, tube formation was notably reduced for wild-type and genetically modified ASCs. This effect could be abrogated, when IGFBP7 was knocked down in ASCs prior to co-culture (Liu F. et al., 2023). Comparably, conditioned medium derived from osteogenically induced MSCs inhibited HUVEC tube formation, sprouting and migration (Sun et al., 2022), while silencing of IGFBP7 expression in these MSCs was able to counter the inhibition. Proteomic analysis indicated that proteins functionally related to cell movement and cellular cytoskeleton organization exhibited significant changes in HUVECs exposed to soluble IGFBP7. Indeed, these HUVECs exhibited less cell polarization, slower migration and impaired tip-cell specification (Sun et al., 2022).

On the other hand, it is essential to note that stromal cells such as MSCs and fibroblasts, as well as their paracrine factors, are generally believed to promote angiogenic processes, while the extent of this bioactivity is influenced by stromal cell heterogenicity (Chang et al., 2002; Jiang et al., 2020), cell culture duration and conditions (Newman et al., 2011). In fact, a high throughput bioassay based on a vasculogenesis model in microfluidic devices demonstrated that MSCs derived from multiple donors at different passages exhibited significant heterogeneity in their ability to support HUVEC lumen formation and maintenance in co-cultures. MSCs’ vasculogenic bioactivity was also positively correlated with the baseline expression of several genes including IGFBP7 (Lam et al., 2022). The validity of these results was further supported by an independent study investigating the influence of immortalized fibroblasts from different passages on lumenogenesis of immortalized HUVECs (Wan et al., 2021). HUVECs could only form functional perfusable microvascular networks with immortalized fibroblasts from earlier passages but not later passages, which correlated with a decrease of Thy1 expression in fibroblasts from later passages. Compared to Thy1^−^ fibroblasts, Thy1^+^ fibroblasts expressed higher IGFBP2, IGFBP7, and secreted protein acidic and rich in cysteine (SPARC). Although the addition of IGFBP7 and SPARC together partially rescued morphological defects in microvascular networks formed with Thy1^-^ fibroblasts, supplementing the combination of all three factors fully rescued those defects. These findings were further confirmed by knocking out IGFBP7 and SPARC individually or together in fibroblasts (Wan et al., 2021).

Consistently, when normal human lung fibroblasts were co-cultured with HUVECs in a 3-dimensional fibrin gel bead assay, robust sprouting and lumenogenesis were observed (Newman et al., 2011). Substituting fibroblasts with fibroblast-derived conditioned medium also promoted endothelial sprouting and lumen formation, although to a lesser extent. In the absence of fibroblasts, a combination of angiopoietin-1 (ANG-1), angiogenin, heypatocyte growth factor (HGF), transforming growth factor-α (TGF-α), and tumor necrosis factor (TNF) drove robust endothelial cell sprouting without the formation of lumina. Subsequent addition of fibroblast-conditioned medium restored lumenogenesis. The study also claimed that combinational knockdown of IGFBP7, collagen I (Col I), procollagen C endopeptidase enhancer 1 (PCOLCE), SPARC and TGF-β–induced protein ig-h3 (βig-h3) in fibroblasts impaired lumen formation significantly, without affecting the sprouting process, whereas individual knockdown of any of the aforementioned proteins did not influence lumenogenesis. The addition of IGFBP7, together with Col I and SPARC, to fibrin gels resulted in increased stiffness of these hydrogels and could rescue the impaired lumen formation in knockdown experiments (Newman et al., 2011). The biomechanical properties of the microenvironment are known to affect angiogenesis. Substrates that are too stiff (Saunders and Hammer, 2010) or too soft (Califano and Reinhart-King, 2008; Guo et al., 2022) can impair angiogenic processes *in vitro* and *in vivo*. Hence, the current studies suggest that IGFBP7 may partially modulate angiogenic processes by modulating matrix stiffness.

Recently, it was even reported that IGFBP7 was embedded in the insoluble ECM of cultured MSCs and that matrices with enhanced pro-angiogenic properties were strongly enriched in IGFBP7, besides other pro-angiogenic components (Spater et al., 2022). Indeed, these MSC-derived ECM-based biologics most significantly facilitated the expression of pro-angiogenic cytokines in macrophages, promoted HUVEC proliferation and sprouting *in vitro,* and enhanced revascularization in mouse full-thickness skin wounds *in vivo* (Spater et al., 2022).

**FIGURE 2:**
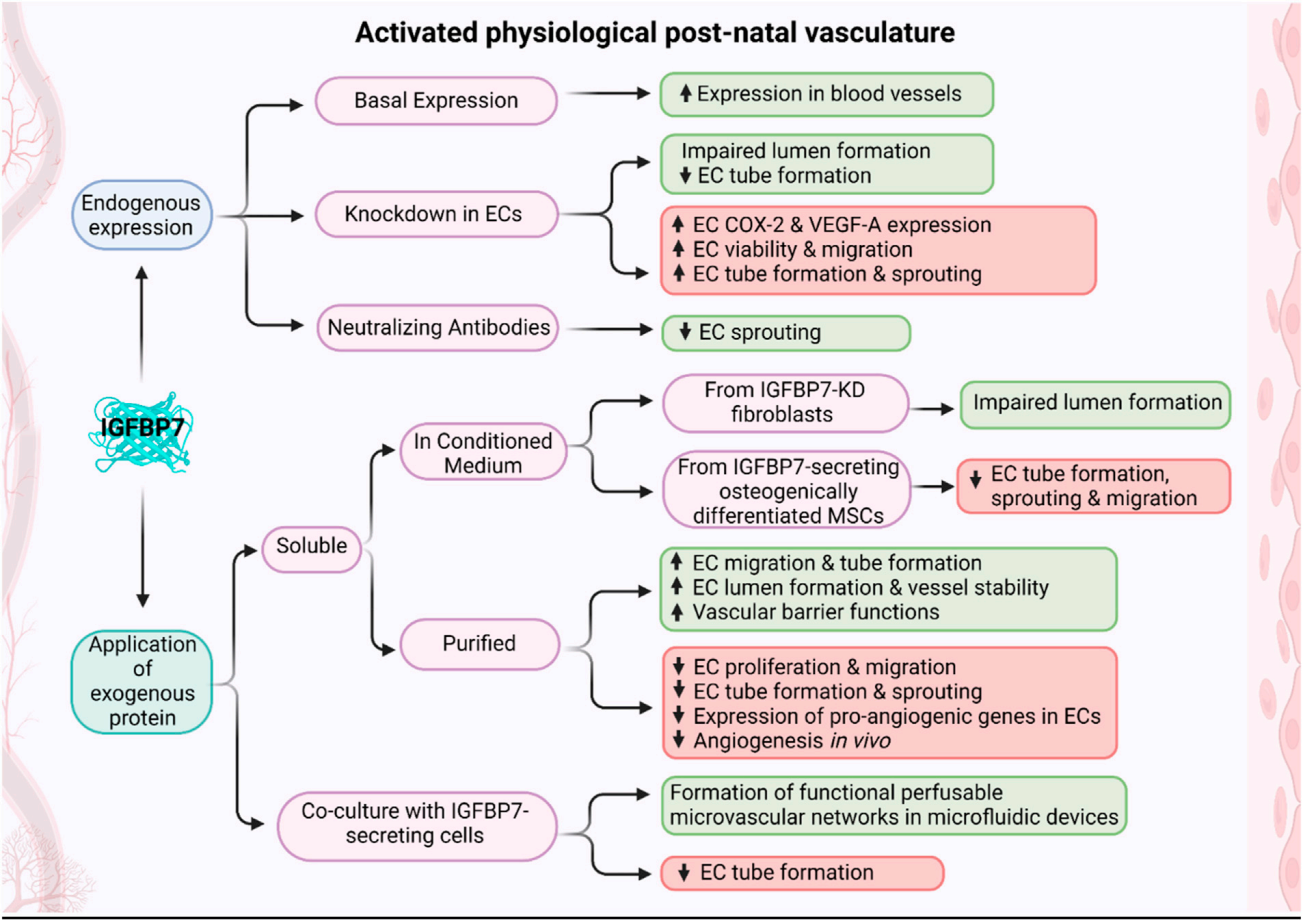
Schematic of effects of IGFBP7 on activated post-natal vasculature. Depending on the experimental design, when IGFBP7 was either added exogenously or its endogenous expression/activity modified, pro-angiogenic (green boxes) or anti-angiogenic (red boxes) properties of IGFBP7 were reported (Schematic was generated using BioRender.com with an academic license).

Although the reported *in vitro* results are inconsistent, at the current stage, there is a strong notion of IGFBP7 being crucial in lumen formation and, thus, in later stages of angiogenesis (Figure 2; Table 2). The role of IGFBP7 in earlier angiogenic stages, such as endothelial cell proliferation, migration, and sprouting remains to be determined (Figure 2; Table 2), especially since the physiological relevance of the performed *in vitro* studies needs to be taken into consideration. Experiments conducted in microphysiological systems, such as vasculogenesis-based assays in microfluidic devices, are more physiologically relevant than tube formation assays on Matrigel (Beyer et al., 2021; Lam et al., 2022; Wan et al., 2023). Here, microvascular network-on-a-chip experiments have shown that IGFBP7 is not acting alone, but in synergy with other secreted ECM proteins (Wan et al., 2021; Lam et al., 2022). Furthermore, current reports on IGFBP7 modulating matrix stiffness in these microphysiological systems point to IGFBP7 being embedded within the microenvironment, thus acting as part of a multifactorial insoluble milieu (Newman et al., 2011). In fact, insoluble MSC-derived ECM enriched in IGFBP7 exhibited strong angiogenic effects *in vitro* and *in vivo* (Spater et al., 2022). This is in stark contrast to studies that utilized exogenous soluble IGFBP7 alone or as part of a soluble secretome in the supernatant of 2-dimensional cultures (Tamura et al., 2009; Tamura et al., 2014; Sun et al., 2022; Liu F. et al., 2023), where IGFBP7 would potentially act outside its inherent context, which may explain the conflicting results. Furthermore, this is also in line with *in vivo* experiments, where the introduction of soluble IGFBP7 resulted in anti-angiogenic properties (Sun et al., 2022), while knockdown experiments in the developing zebrafish (Hooper et al., 2009) and high expression of IGFBP7 in angiogenic developing (Hooper et al., 2009; Bar et al., 2020) and adult vasculature (Hooper et al., 2009; Buga et al., 2014; Wang et al., 2021) pointed to pro-angiogenic properties of IGFBP7. Indeed, soluble IGFBP7 may even compete with ECM-bound IGFBP7 for receptor binding, resulting in opposite or inhibitory effects. Overall, bioactivities of IGFBP7, while embedded in its integral microenvironment, are believed to be more physiologically pertinent.

**TABLE 2:** Role of IGFBP7 in activated physiological post-natal vasculature.

Administration/Experimental approach	Results	Potential effects on the vasculature	References
Endogenous expression	• Increased expression in the vasculature of regenerating skin in a mouse model • Increased expression in vasculature in an oxygen-induced retinopathy model of angiogenesis in mice • Increased expression in bone marrow sinusoidal vessels after 5-fluorouracil-induced angiogenesis in mice	Pro-angiogenic	Hooper et al. (2009)
Endogenous expression	• Increased expression in the vasculature of patients with post-traumatic brain injury and stroke	Pro-angiogenic	Buga et al. (2014), Wang et al. (2021)
Dosing with exogenous soluble protein	• Local injection abolished VEGF-A induced vessel hyperpermeability in mice	Enhances vascular barrier functions	Bar et al. (2020)
Dosing with exogenous soluble protein	• Reduced tube formation, spheroid sprouting, and expression of tip-cell-enriched genes in HUVECs • Intraocular injection resulted in inhibition of retinal angiogenesis in mice • Early injection into calvarial bone defect impaired angiogenesis in mice	Anti-angiogenic	Sun et al. (2022)
Dosing with exogenous soluble protein	• Inhibited VEGF-A induced proliferation and tube formation of HUVECs	Anti-angiogenic	Tamura et al. (2009)
Dosing with exogenous soluble protein	• Inhibited LH-induced proliferation, migration and tube formation of rat LECs	Anti-angiogenic	Tamura et al. (2014)
Dosing with exogenous soluble protein	• Reduced tube formation of HUVECs	Anti-angiogenic	Liu et al. (2023a)
Knockdown of endogenous expression in ECs	• Knockdown enhanced COX-2 and VEGF-A expression by HUVEC	Anti-angiogenic	Tamura et al. (2009)
Knockdown of endogenous expression in ECs and rescue by dosing with exogenous soluble protein	• Knockdown promoted cell viability, migration, increased tube length, and VEGF-A expression of Rhesus Monkey RF/6A choroidal ECs • Addition of soluble IGFBP7 attenuated this increased VEGF-A expression	Anti-angiogenic	Zhu et al. (2020)
Knockdown of endogenous expression in ECs	• Knockdown inhibited tube formation of HUVECs	Pro-angiogenic	Sun et al. (2021)
Dosing with exogenous soluble protein	• Promoted tube formation and migration of HUVECs • Knockdown of IGFBP7 receptor CD93 in HUVECs attenuated IGFBP7’s ability to promote tube formation and migration	Pro-angiogenic	Sun et al. (2021)
Neutralizing antibody against IGFBP7	• Inhibited sprouting of bovine capillary ECs	Pro-angiogenic	Van Beijnum et al. (2006)
Dosing with exogenous soluble protein	• Promoted tube formation of human brain EC in Matrigel	Pro-angiogenic	Pen et al. (2008)
Supplementation of IGFBP7-secreting cells in co-culture	• HUVEC tube formation was reduced when co-cultured with wild-type and genetically modified ASC overexpressing IGFBP7; the reduction was abrogated when IGFBP7 was knocked down in ASCs	Anti-angiogenic	Liu et al. (2023a)
Conditioned medium from IGFBP7 secreting cells	• Inhibited tube formation, sprouting and migration of HUVECs when cultured in conditioned medium from osteogenically differentiated MSCs; effect was abrogated when IGFBP7 was knocked down in MSCs	Anti-angiogenic	Sun et al. (2022)
Dosing with exogenous soluble protein	• Slower migration and impaired tip-cell specification of HUVECs	Anti-angiogenic	Sun et al. (2022)
Supplementation of IGFBP7 secreting cells in co-culture	• Formation of functional perfusable microvascular networks by HUVECs in co-culture with fibroblasts in microfluidic devices; Knockdown of IGFBP7 in the co-cultured fibroblasts inhibited formation of functional perfusable microvascular networks	Pro-angiogenic, promotes lumen formation and vessel stability	Wan et al. (2021)
Dosing with exogenous soluble protein	• Partially rescue of functional perfusable microvascular networks by HUVECs in microfluidic devices, when co-cultured with fibroblasts that did not support lumen formation and vessel maintenance • Combination of soluble IGFBP2, IGFBP7 and SPARC fully rescued the formation of functional perfusable microvascular networks by HUVEC in microfluidic device when co-cultured with fibroblasts that did not support lumen formation and vessel maintenance	Pro-angiogenic, promotes lumen formation and vessel stability	Wan et al. (2021)
Conditioned medium from IGFBP7 secreting cells and rescue by dosing with exogenous soluble protein	• Impaired lumen formation by HUVECs when cultured in conditioned medium from fibroblasts with combinational knockdown of IGFBP7, Col I, POLCE, SPARC and βig-h3 • Addition of IGFBP7 alone was not sufficient to rescue lumen formation. However, the combination of IGFBP7, Col I, and SPARC was able to fully rescue the defects	Pro-angiogenic	Newman et al. (2011)
Insoluble ECM enriched with IGFBP7	• Promoted expression of pro-angiogenic cytokines in macrophages • Increased proliferation of HUVECs, and sprout length of HUVEC spheroids • Promoted blood vessel in-growth into full-thickness cutaneous wounds in healthy mice	Pro-angiogenic	Spater et al. (2022)

### IGFBP7 in tumor vasculature formation

The aggressive growth of cancer cells and the associated overexpression of pro-angiogenic factors often lead to the development of disorganized blood vessel networks that are immature, tortuous, hyperpermeable and fundamentally different from normal vasculature (Siemann, 2011). The role of IGFBP7 in pathological tumor vasculature is thus reviewed separately.

**FIGURE 3:**
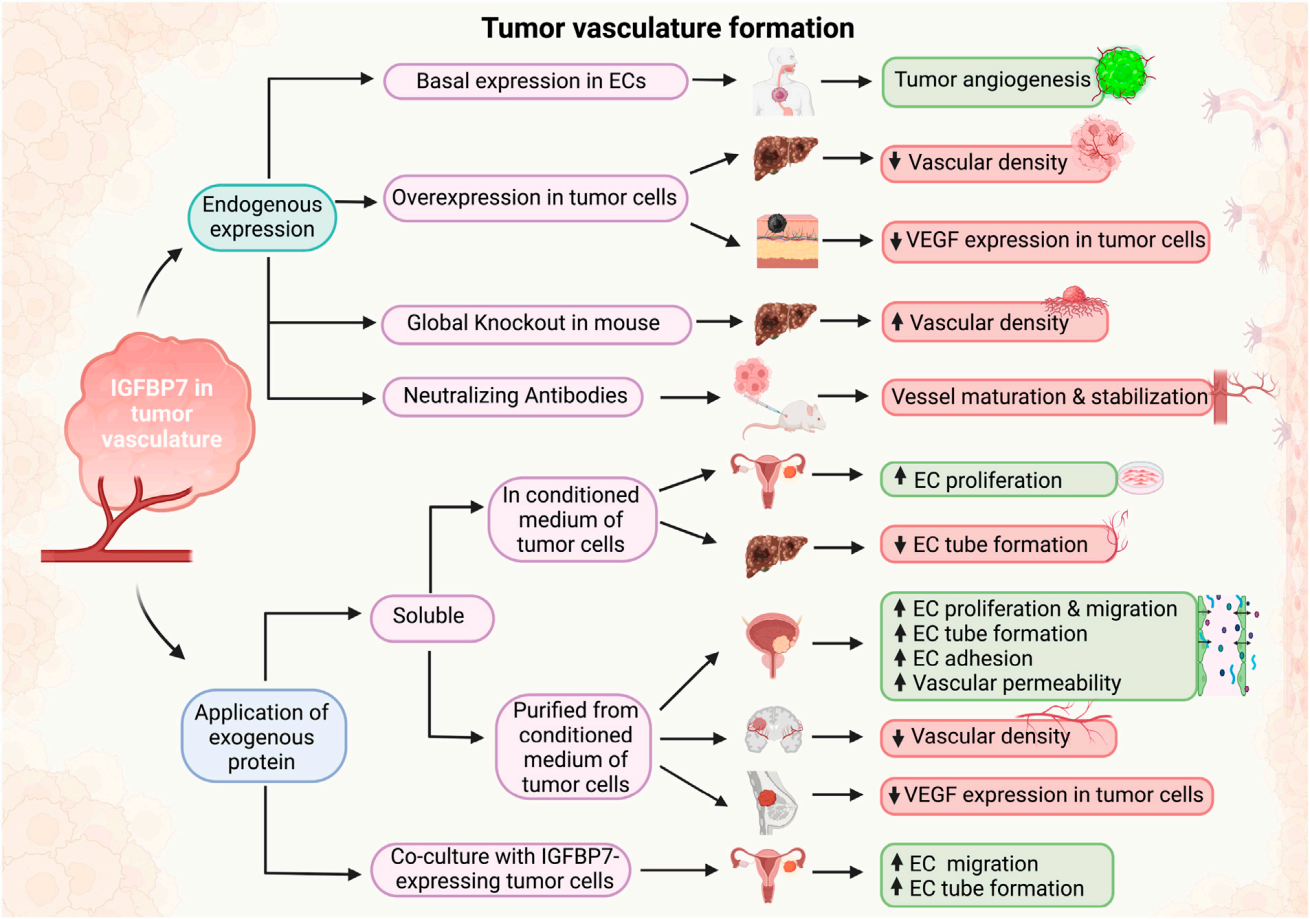
Schematic of effects of IGFBP7 in tumor vasculature formation. Depending on the experimental design, when IGFBP7 was either added exogenously or its endogenous expression/activity modified, pro-angiogenic (green boxes) or anti-angiogenic (red boxes) properties of IGFBP7 were reported (Schematic was generated using BioRender.com with an academic license).

Within the tumorigenesis context, IGFBP7 was suggested to function as a “double-edged sword” in cancer progression, acting as an oncogene in some and as a tumor-suppressor in other cancer types (Jin et al., 2020). Likewise, an ambiguous role of IGFBP7 was reported in tumor angiogenesis. Vascularization is a critical process in tumor growth and is therefore investigated as a promising therapeutic target (Li Y. et al., 2023). The IGFBP7 receptor CD93 has been identified as one of the top 20 genes in tumor angiogenesis. Studies have shown that tumor development in CD93 knockout mice or loss of CD93 in endothelial cells were associated with disruption of endothelial junctions and increased vascular permeability. In contrast, it was also demonstrated that the IGFBP7-CD93 axis was related to disordered tumor vasculature and that CD93 neutralizing antibody could normalize tumor vasculature. The authors illustrated that neutralizing antibodies against IGFBP7 administered to tumor-bearing mice revealed no differences in blood vessel density, but improved pericyte coverage, indicative of vessel maturation (Sun et al., 2021). Blockade of the CD93/IGFBP7 interaction by monoclonal antibodies thus promoted vascular maturation and reduced leakage, leading to reduced tumor hypoxia and increased tumor perfusion (Figure 3; Table 3). This promoted drug delivery, resulting in an improved antitumor response to chemo- and immuno-therapy (Sun et al., 2021).

**TABLE 3:** Role of IGFBP7 in pathological vasculature formation.

Administration/Experimental approach	Results	Potential effects on the vasculature	References
Neutralizing antibody against IGFBP7	• Intraperitoneal injection promoted maturation of blood vessels within tumor xenografts in mice	Vessel maturation and stabilization	Sun et al. (2021)
Dosing with exogenous soluble protein isolated from conditioned media of IGFBP7 secreting tumor cells	• IGFBP7 isolated from conditioned media of human bladder carcinoma cells did not stimulate growth or migration, but enhanced adhesion of HUVECs when protein was coated on to cell culture plate • Promoted the retraction of HUVECs by inducing actin stress fibers and loosening their VE-cadherin-mediated intercellular junction • Increased permeability of HUVEC monolayer • Intradermal injection increased vessel permeability near the injection site in mice	Promoted endothelial cell adhesion and increased permeability	Komiya et al. (2014)
Dosing with exogenous soluble protein isolated from conditioned media of IGFBP7 secreting tumor cells	• Protein isolated from conditioned media of human bladder carcinoma cells promoted adhesion of ECV-304 HUVEC on Col IV coated surfaces • Promoted endothelial tube formation	Promoted endothelial cell adhesion Pro-angiogenic	Akaogi et al. (1996a)
Overexpression in tumor cells	• Reduced vessel growth into HCC tumor masses overexpressing IGFBP7 in CAM assay	Anti-angiogenic	Chen et al. (2011)
Conditioned medium from tumor cells overexpressing IGFBP7	• Reduced tube formation by HUVECs, when cultured in conditioned medium from HCC cells overexpressing IGFBP7	Anti-angiogenic	Chen et al. (2011)
Overexpression in tumor cells	• Reduced CD31^+^ vessel density in HCC xenografts overexpressing IGFBP7	Anti-angiogenic	Chen et al. (2011)
Knockout mutant	• Increased CD31 staining in HCC tissues of IGFBP7−/− mice	Anti-angiogenic	Akiel et al. (2017)
Dosing with exogenous soluble protein	• GBM tumors with reduced vascular density formed in CAM assay	Anti-angiogenic	Pen et al. (2011)
Dosing with exogenous soluble protein	• Intravenous and intratumoral injection reduced vascular density and VEGF expression in breast cancer xenografts in mice	Anti-angiogenic	Benatar et al. (2012)
Endogenous expression	• Highly expressed by CECs in human patients with metastatic carcinomas, where number of CECs is correlated with degree of tumor angiogenesis	Pro-angiogenic	Smirnov et al. (2006)
Conditioned medium from IGFBP7 secreting tumor cells	• Enhanced proliferation of HOMECs when cultured in a conditioned medium from EOC	Pro-angiogenic	Winiarski et al. (2013)
Supplementation by IGFBP7 secreting cells in co-culture	• Enhanced migration and tube formation of HOMECs when co-cultured with EOC	Pro-angiogenic	Winiarski et al. (2013)
Dosing with exogenous soluble protein	• Promoted proliferation, migration and tube formation by HOMEC	Pro-angiogenic	Winiarski et al. (2013)
Overexpression in tumor cells	• Reduced VEGF expression in B6-F10 melanoma cells overexpressing IGFBP7	Anti-angiogenesis	Chen et al. (2010b)

Integrin αvβ3 was identified as another receptor of IGFBP7 in HUVECs. When isolated from conditioned medium of human bladder carcinoma cells and coated on cell culture plates, IGFBP7 did not stimulate the growth or migration of HUVECs, but enhanced their efficient adhesion (Komiya et al., 2014). This result was consistent with a previous study showing that IGFBP7 facilitated ECV-304 HUVEC attachment to Col IV (Akaogi et al., 1996a). IGFBP7 was also implicated in the formation of tube-like structures of endothelial cells in collagen I hydrogels, while these effects could be abolished when exogenous heparin was added to compete with the binding of IGFBP7 to cell surface heparan sulfate (Akaogi et al., 1996a). IGFBP7 also promoted the retraction of endothelial cells by inducing actin stress fibers and loosening their VE-cadherin-mediated intercellular junction. Consequently, IGFBP7 increased vascular permeability both *in vitro* and *in vivo* (Komiya et al., 2014) (Figure 3; Table 3).

IGFBP7 expression was significantly downregulated in human hepatocellular carcinoma (HCC) tissue samples and cell lines as compared with normal liver and hepatocytes, respectively. Forced overexpression of IGFBP7 in HCC cells induced senescence, and profoundly suppressed *in vivo* tumor growth. When HCC cells overexpressing IGFBP7 were implanted in a chicken chorioallantoic membrane (CAM) assay, angiogenesis was significantly reduced in the formed tumor masses. Similarly, HUVECs exposed to a conditioned medium from these cells exhibited reduced tube formation on Matrigel (Chen et al., 2011). Hence, reduced tumor growth may at least be partially attributed to reduced revascularization due to IGFBP7 overexpression. Increased CD31 staining in hepatocarcinoma tissues was also noted in IGFBP7^−/−^ mice, which were reported to be more susceptible to developing tumors (Akiel et al., 2017). Congruently, xenografts of human HCC HepG3 cells overexpressing IGFBP7 showed downregulation in vessel density (Chen et al., 2011) (Figure 3; Table 3).

Exogenously applied IGFBP7 also reduced proliferation and induced senescence in glioblastoma (GBM) cell lines. When implanted in a CAM assay, GBM cells formed solid and highly vascularized tumors that were reduced when treated with soluble IGFBP7. Vessels in IGFBP7-treated tumors were clustered, unevenly distributed, and associated with a higher number of perivascular cells (Pen et al., 2011). Systematically administered IGFBP7 into mice with breast cancer cell line xenografts also resulted in reduced vessel density in xenografts sections (Benatar et al., 2012) (Figure 3; Table 3).

In other tumors, hypoxia, VEGF-A and TGF-β1 were associated with the expression of IGFBP7 in vascular endothelial cells, and indeed, IGFBP7 was described to be expressed at much higher levels in some tumors as compared to non-carcinogenic tissues (Pen et al., 2007; 2008; Hooper et al., 2009). In patients with metastatic carcinomas, IGFBP7 was reported to be highly expressed in circulating endothelial cells (CECs) (Smirnov et al., 2006), where the number of CECs correlated with the degree of tumor angiogenesis (Shaked et al., 2005). The proliferation of human ovarian microvascular endothelial cells (HOMECs) was noted to be enhanced when the cells were treated with a conditioned medium derived from epithelial ovarian cancer (EOC). Secretomics identified IGFBP7 as one of the enriched factors within the conditioned medium. When co-cultured with EOC cells, HOMECs exhibited enhanced migration in a transwell assay, as well as tube formation. Likewise, the addition of exogenous IGFBP7 promoted the proliferation, migration, and tube formation of HOMECs (Winiarski et al., 2013).

The tumor microenvironment (TME) plays a decisive role in tumor progression and angiogenesis. Hence, IGFBP7 may also affect these processes by modulating the TME (Xiuqing et al., 2022). Indeed, IGFBP7 was reported to be highly expressed in the stroma of some tumors and co-localized with activated cancer-associated fibroblasts (CAFs). *In vitro* analysis indicated that TGF-β1 can act as an important inducer of IGFBP7 expression in fibroblasts. IGFBP7, purified from the conditioned medium from bladder carcinoma cells, stimulated fibronectin and α-smooth muscle actin (α-SMA) synthesis in normal fibroblasts, as well as promoted fibroblast proliferation and migration. A transforming growth factor-β (TGF-β) signal inhibitor attenuated IGFBP7-induced expression of fibronectin and α-SMA, but did not affect IGFBP7’s stimulation of cell growth and migration, suggesting TGF-β dependent and independent mechanisms for IGFBP7-driven CAF activation, which is known to contribute to tumor progression (Komiya et al., 2012). Similar results were recently confirmed in gastric cancer, where IGFBP7 was mainly expressed by myofibroblastic CAFs (Hong et al., 2023). TGF-β signaling promoted the expression of IGFBP7 in CAFs, which in turn enhanced fibroblast growth factor-2 (FGF-2) expression in gastric cancer and resulted in increased macrophage infiltration and poor prognosis. Treatment of macrophages and gastric cancer cells with recombinant IGFBP7 further showed that IGFBP7 promoted tissue-associated macrophages (TAM)/M2 polarization *via* FGF-2/Fibroblast Growth Factor Receptor 1 (FGFR-1)/Phosphoinositide 3-kinases (PI3K)/Protein kinase B (Akt) axis (Li D. et al., 2023). Activation of CAFs and abundance of TAMs with a M2 phenotype are associated with enhanced angiogenesis and tumor progression (Wang et al., 2019).

Vascular permeability is an indicator of activated angiogenic vasculature (Dvorak H F et al., 1995) and this process is often dysregulated in tumors (Tomita et al., 2021). In contrast, increased vascular barrier functions indicate quiescent and mature vasculature (Vandoorne et al., 2010; Wan et al., 2023). IGFBP7 has been observed to be overexpressed in some tumors (Pen et al., 2007; Hooper et al., 2009; Liu Y. et al., 2023; Xu et al., 2023), while repressed in others (Murphy et al., 1993; Liu Y. et al., 2023; Xu et al., 2023). Similarly, IGFBP7 has been suggested to increase vascular permeability in some tumors, while decreasing it in others. It is noteworthy that the knockdown of IGFBP7 increased permeability, while its partial inhibition by neutralizing antibodies decreased permeability (Sun et al., 2021). The observed differences may be related to the degree of IGFBP7-based signaling being silenced in tumors, where its ablation led to vascular dysfunction, while partial neutralization may have downregulated its overactivation and thus normalized vasculature. For the inconsistent findings on vascular permeability upon application of soluble IGFBP7 (Komiya et al., 2014; Sun et al., 2022), it has to be taken into consideration that IGFBP7 acted outside its inherent microenvironment, potentially resulting in different bioactivity. This is further supported by the notion that integrin αvβ3 acts as a receptor for IGFBP7 and that the interaction of cells with immobilized IGFBP7 resulted in cytoskeletal reorganization (Komiya et al., 2014), suggesting that IGFBP7 may function *via* mechanotransduction, thus requiring IGFBP7 to be immobilized in the ECM for relevant signal transduction.

IGFBP7 expression is not restricted to the tumor vasculature but can be expressed by tumor cells, as well as stromal cells in the TME. Since it has been implicated by studies of physiological angiogenesis that IGFBP7 acts in synergy with other ECM components (Newman et al., 2011; Wan et al., 2021; Lam et al., 2022) and that IGFBP7’s bioactivity is dependent on the microenvironment, conflicting results observed in tumor angiogenesis and progression may be explained by differences in the composition of the TME in which IGFBP7 is embedded in. Furthermore, IGFBP7 may exert different functions in various cell types, with IGFBP7 signaling in CAFs or tumor vasculature overriding signaling in tumor cells and *vice versa* (Rupp et al., 2015; Li Y. et al., 2023). Hence, the indirect effects of IGFBP7 on tumor vasculature must also be considered, where IGFBP7 signaling on tumor cells may enhance or reduce their expression of pro-angiogenic factors (Chen R.-Y. et al., 2010; Benatar et al., 2012; Akiel et al., 2017; Zhu et al., 2020). Indeed, overexpression of IGFBP7 in B16-F10 melanoma cells downregulated the expression of VEGF (Chen R.-Y. et al., 2010). Congruently, increased VEGF levels were recorded in lung tumors of IGFBP7^−/−^ mice (Akiel et al., 2017) and administration of exogenous IGFBP7 reduced VEGF expression in breast cancer cell lines 1833 xenografts implanted in mice (Benatar et al., 2012).

## Ligand binding and regulation of down-stream signaling

To further understand the function of IGFBP7, its interaction with known ligands and downstream effects on signaling activation need to be examined.

Soluble IGFBP7 was shown to bind IGF-I in an immunoblotting assay (Oh et al., 1996). Treatment of mouse fibroblasts with soluble IGFBP7 promoted their proliferation, while the pro-proliferative effect of IGFBP7 was enhanced when IGF-I was added to cultures (Akaogi et al., 1996b). In acute lymphoblastic leukaemia (ALL) cells, soluble IGFBP7 was observed to synergistically activate the IGF-I receptor with IGF-I (Chen R.-Y. et al., 2010). These studies suggested that IGFBP7 could enhance IGF-I binding to its receptor and thus promote IGF signaling.

Nonetheless, IGFBP7, which contains an IGF-I receptor binding motif, was also reported to block IGF binding to its receptor and thus prevent the activation of downstream IGF-I receptor/Akt/Serine/threonine-protein kinase mTOR (mTOR)/Eukaryotic translation initiation factor 4E-binding protein 1 (4E-BP1) pathway in breast cancer cells. This suppressed expression of genes associated with cancer cell proliferation (Evdokimova et al., 2012). Another study in IGFBP7^−/−^ mice partially supported these findings, as constitutive activation along the IGF-I receptor/Akt/Glycogen synthase kinase-3 beta (GSK-3 beta) axis was observed in hepatocytes and embryonic fibroblasts (Akiel et al., 2017).

IGFBP7 was also shown to bind insulin (Oh et al., 1996), and soluble IGFBP7 and insulin synergistically enhanced the proliferation of mouse fibroblasts (Akaogi et al., 1996b). In another study, it was reported that soluble IGFBP7 and insulin synergistically activated insulin receptor/Akt and/ERK1/2 pathways to promote the proliferation of ALL cells. IGFBP7 binding to the IGF-I receptor was also reported to protect the receptor from internalization, preventing it from lysosomal degradation and thus prolonging insulin-induced responses in stimulating the proliferation of ALL cells. Interestingly, IGFBP7 and insulin synergistically prolonged the activation of the IGF-I receptor, but not the insulin receptor (Artico et al., 2021). On the other hand, it was also suggested that binding of soluble IGFBP7 to insulin blocked insulin-stimulated insulin receptor activation in NIH-3T3 fibroblasts (Yamanaka et al., 1997). Other reports indicated that endogenously expressed IGFBP7 had no significant effects on insulin signaling in the IGFBP7-expressing human bladder carcinoma cell line EJ-1 and the human colon cancer cell line DLD-1 transfected with the IGFBP7 gene (Sato et al., 2007).

In contrast to other members of the IGFBP family, IGFBP7 exhibits a strong homology to follistatin (Kato, 2000). Similar to follistatin, IGFBP7 was found to bind activin A (Kato, 2000) and when supplemented to cultures, neutralized the activity of activin to inhibit osteogenesis in human bone marrow MSCs (Bolomsky et al., 2015), and promote steroidogenesis in rat ovarial granulosa cells (Tamura et al., 2007).

IGFBP7 also contains a heparin-binding motif (Sato et al., 1999; Evdokimova et al., 2012) and heparan sulfate is involved in the interaction of cells with IGFBP7. Indeed, when ECV-304 HUVECs and BALB/c3T3 mouse fibroblasts were treated with heparinase their ability to adhere to IGFBP7-coated plates was abolished (Sato et al., 1999). Furthermore, heparan sulfate was implicated in modulating IGFBP7’s activity. In fact, a truncated IGFBP7 protein, lacking the heparan sulfate binding motif abolished the binding of IGFBP7 to the IGF-I receptor (Evdokimova et al., 2012).

Other ligands of IGFBP7 are chemokines such as CCL5, CCL21 and CXCL10. It was suggested that IGFBP7 can bind those factors, while embedded in the basal lamina of blood vessels and present them to trafficking lymphocytes (Nagakubo et al., 2003). Congruently, IGFBP7 was shown to bind VEGF-A, when immobilized on a surface (Usui et al., 2002), while unable to bind VEGF, when added in its soluble form into cell culture supernatant (Tamura et al., 2009), suggesting again that IGFBP7 may exert distinct functions when properly embedded in its microenvironment. This is in line with studies on IGFBP7 modulating VEGF signaling, which reported conflicting results (Hooper et al., 2009; Tamura et al., 2009; Tamura et al., 2014; Sun et al., 2022).

Current results clearly indicate that IGFBP7 can act in an IGF-dependent and -independent manner. It can also modulate the signaling of other ligands, including VEGF (Usui et al., 2002), insulin (Oh et al., 1996) and activin (Kato, 2000), although the reports on resulting effects are conflicting, suggesting again that IGFBP7’s bioactivity may be context-dependent. IGFBP7’s ability to directly bind other cytokines and chemokines also opens the avenue of IGFBP7 acting as a signaling factor reservoir, where it can bind sequester, and/or present them to cells. Notably, IGFBP7 ligands such as VEGF (Leung et al., 1989), IGFs (Nakao-Hayashi et al., 1992; Lee et al., 2000), insulin (Nakao-Hayashi et al., 1992) and activin (Kaneda et al., 2011; de Oliveira et al., 2020) were implicated in angiogenic processes. The here-described signaling pathways may thus also be relevant in the modulation of angiogenic processes by IGFBP7.

## IGFBP7 in stem cell commitment

Besides its crucial role in vasculature development, IGFBP7’s involvement in stem and progenitor cell commitment has been observed in adult and embryonic cells. During the differentiation of bovine pre-adipocytes into adipocytes, IGFBP7 was significantly upregulated. IGFBP7 gene overexpression and RNA interference promoted lipid accumulation and inhibited triglyceride production in mature adipocytes, respectively, suggesting that IGFBP7 may play a regulatory role in adipocyte commitment (Hu et al., 2021).

During the differentiation of human MSCs along the osteogenic lineage (Wan et al., 2022), overexpression of IGFBP7 enhanced the expression of osteo-specific genes and proteins, while IGFBP7 knockdown decreased osteogenesis-specific markers. Researchers demonstrated that IGFBP7 promoted osteogenic differentiation *via* the Wnt/β-catenin signaling pathway. Using a rat tibial osteotomy model, a sheet of IGFBP7–overexpressing MSCs improved bone healing over MSC-sheets with unmodified IGFBP7 expression levels (Zhang et al., 2018).

Intermittent administration of parathyroid hormone (PTH), an anabolic agent approved for the treatment of osteoporosis, was found to increase the expression of IGFBP7 in mouse MSCs and pre-osteoblasts. Anabolic effects of PTH were interrupted upon knockdown of IGFBP7 and supplementation of IGFBP7 could enhance the bone-forming efficacy of PTH, suggesting that IGFBP7 was involved in the bone-forming effects of PTH. Researchers also suggested that IGFBP7 may inhibit the mTOR pathway and enhance TGF-β/bone morphogenetic protein (BMP) and Wnt-β-catenin signaling cascades during osteogenic differentiation (Xia et al., 2022). Indeed, this was supported by other studies, where treatment with IGFBP7 was shown to upregulate the expression of BMP2 in MSCs (Xia et al., 2022) and fibroblasts (Lu et al., 2020). *In vivo*, bone healing was accelerated by the intravenous administration of IGFBP7 along with PTH in a mouse tibia fracture model (Xia et al., 2022).

Another study showed that IGFBP7 expression and secretion gradually increased during osteogenic differentiation of bone marrow-derived-MSCs, while during cranial bone defect healing IGFBP7 peaked at 2 weeks. Injection of soluble IGFBP7 into the defect side at day 4 of healing, potentially shifting this peak to an earlier time point, inhibited neo-vasculature in the defect region and reduced bone formation (Sun et al., 2022). Although the experiments were again performed using soluble IGFBP7, thus not within its inherent microenvironment, current results suggest that while IGFBP7 is important, coordination of temporal dosing is crucial for proper bone healing.

IGFBP7 was also found to be necessary for cardiac commitment of mouse embryonic stem cells (ESCs) and its regulation depended on the transcription factor TAp63, a p63 isoform. TAp63 directly activated both IGFBP7 and Activin-A during ESC cardiogenesis while these secreted factors modulated TAp63 gene expression by a feedback loop mechanism (Wolchinsky et al., 2014).

Collectively, current results suggest that IGFBP7 is involved in progenitor and stem cell commitment. Nonetheless, the amount of evidence is still relatively limited and future studies are needed to understand the role of IGFBP7 in cell differentiation processes.

## IGFBP7 in tissue remodeling

IGFBP7 is also implicated in physiological and pathological tissue remodeling, including muscle hypertrophy, wound healing, as well as physiological and pathological fibrosis.

Besides being upregulated in the activated vasculature (Van Beijnum et al., 2006; Pen et al., 2008; Hooper et al., 2009; Tamura et al., 2009; Tamura et al., 2014; Abu-Safieh et al., 2011; Buga et al., 2014; Komiya et al., 2014; Bar et al., 2020; Zhu et al., 2020; Sun et al., 2021; Sun et al., 2022; Wan et al., 2021; Wang et al., 2021; Liu F. et al., 2023), IGFBP7 was reported also to modulate other processes and affect other cell types during wound healing. *In vitro*, IGFBP7-containing conditioned medium from MSCs, as well as recombinant IGFBP7 enhanced the migration of human small airway epithelial cells, suggesting that IGFBP7 may be involved in the MSC-mediated lung injury repair (Akram et al., 2013). IGFBP7 was also expressed in keratinocytes and the knockdown of IGFBP7 in these cells resulted in decreased susceptibility to TNFα-induced apoptosis but did not affect senescence. IGFBP7 silencing also downregulated genes associated with calcium-induced differentiation of keratinocytes (Severino et al., 2013), suggesting that IGFBP7 may also be involved in the re-epithelialization of skin wounds.

During dysregulated skin wound healing, such as keloid formation, IGFBP7 has been described to have inhibitory effects. Indeed, IGFBP7 expression was decreased in keloid tissues. Treatment of keloid fibroblasts with recombinant IGFBP7 *in vitro* decreased their proliferation and induced apoptosis, while their migration was unaffected. Analysis of the keloid fibroblast transcriptome and protein levels upon IGFBP7 treatment revealed decreased levels of TGF-β1, Col I, VEGF, and pro-inflammatory markers interleukin-6 (IL-6) and interleukin-8 (IL-8), while levels of TGF-β3 were increased. Similarly, co-culture of ASCs, known to express IGFBP7, inhibited keloid fibroblast proliferation, while their migration or apoptosis was unaffected. Knockdown of IGFBP7 in ASCs was able to mitigate the observed anti-proliferative effect. Congruently, ASCs were able to inhibit collagen I production in keloid fibroblasts, while there was no change when co-cultured with IGFBP7-silenced ASCs. Hence, current data suggest that IGFBP7 may have anti-inflammatory and anti-fibrotic properties during dysregulated wound healing. Moreover, the authors concluded that IGFBP-7 secreting ASCs inhibited keloid formation *via* the BRAF/MAPK/ERK signaling pathway (Liu F. et al., 2023).

In contrast, increased expression of IGFBP7 has been observed in patients with non-alcoholic fatty liver disease (NAFLD), which could be reproduced in a zebrafish model. IGFBP7 knockout significantly decreased lipid accumulation, inflammation, and liver fibrosis, whereas liver-specific IGFBP7 overexpression dramatically exacerbated liver fibrosis in the zebrafish NAFLD model, suggesting that IGFBP7 may act as an important regulator in NAFLD progression (Wang et al., 2023).

IGFBP7 was also found to be secreted by liver macrophages and regulate liver metabolism thereby contributing to insulin resistance. It was reported that obesity did not induce pro-inflammatory polarization of liver macrophages from mice and humans and obesity-induced insulin resistance occurred independently of liver macrophage activation. Nonetheless, liver macrophages from mice fed with a high-fat diet exhibited major changes in their transcriptome with IGFBP7 being one of the most upregulated genes. IGFBP7 knockdown in liver macrophages resulted in an upregulation of pro-inflammatory markers in these cells, while there was no major impact on inflammatory gene expression and immune cell content in the overall liver. Hence, IGFBP7 was suggested to exhibit anti-inflammatory or even immunomodulatory properties in liver macrophages. Moreover, IGFBP7 silencing in insulin-resistant animals improved glucose homeostasis, suggesting a detrimental role for IGFBP7 in metabolically impaired animals. Mechanistically, it was shown that IGFBP7 bound to the insulin receptor, enhanced insulin activation of Akt and induced lipogenesis and gluconeogenesis *via* activation of ERK signaling, confirming its direct involvement in insulin metabolism (Morgantini et al., 2019).

The immunomodulatory properties of IGFBP7 were also demonstrated in an experimental colitis model (Liao et al., 2016). IGFBP7 knockdown in MSCs significantly decreased their immunomodulatory properties, decreasing the antiproliferative functions of MSCs against T-cells, while also affecting the pro-inflammatory cytokine production of the T-cells *in vitro*. In a mouse experimental colitis model, intraperitoneally injected MSCs ameliorated the clinical and histopathological severity of induced colonic inflammation and restored the injured gastrointestinal mucosal tissues, while MSCs that were knocked down for IGFBP7 had minimal effects (Liao et al., 2016).

In addition to the anti-inflammatory effects, IGFBP7 was implicated in the protective effects of exercise on proliferating muscle satellite cells. Exercise is known to be beneficial for skeletal muscle homeostasis and regeneration, which is crucial as exhaustion of muscle satellite cells is correlated with muscle diseases. IGFBP7 was upregulated in satellite cells of exercising mice, leading to the inhibition of Akt phosphorylation, mTOR activity, and reduced mitochondrial metabolism, suggesting that exercise protected proliferative satellite cells against exhaustion *via* the IGFBP7/Akt/mTOR axis (Chen et al., 2020).

IGFBP7 has been described to be secreted by failing cardiomyocytes in advanced heart failure (Ko et al., 2022). In a pressure overload mouse heart failure model, IGFBP7 deficiency attenuated cardiac dysfunction by reducing cardiac inflammatory injury, tissue fibrosis, and cellular senescence. Antibody-mediated IGFBP7 neutralization reversed IGFBP7-induced suppression of Forkhead box O3 (FOXO3a), thereby restoring DNA repair and reactive oxygen species detoxification signals and attenuated heart failure in mice (Zhang et al., 2022). IGFBP7 also exhibited increased transcription levels in senescent human mammary epithelial cells (Chen et al., 2020) and MSCs (Severino et al., 2013), while being an integral part of the secretome of senescent MSCs that could induce senescence in young MSCs (Severino et al., 2013). Nonetheless, another study reported that IGFBP7 was able to protect MSCs from senescence and identified the underlying mechanism as the reduction of p21 transcription (Li et al., 2022).

Although conflicting results are reported, IGFBP7 appears to exhibit cell-protective, anti-inflammatory, immunomodulatory, and anti-fibrotic properties. Nonetheless, IGFBP7’s function may be affected by its microenvironment, as elaborated above, and it may exhibit opposite biological functions in strongly dysregulated environments, such as those found in NAFLD and heart failure.

## Conclusion

Decades of research investigating the role of IGFBP7 in tissue (re-)vascularization confirmed that IGFBP7 indeed plays a substantial role in physiological and pathological angiogenesis, however its exact function is difficult to predict. IGFBP7 is highly expressed in activated developing, healing and remodeling vasculature, while its role in these processes is highly ambiguous. It is important to consider that the observed effects on angiogenesis are starkly different, when IGFBP7 is studied in its inherent context, namely, embedded within the native ECM, as compared to studies that supplemented soluble IGFBP7. Indeed, IGFBP7 is an ECM component, while the ECM is composed of a plethora of macromolecules that are assembled into a complex and precise network (Assunção et al., 2020b; Assunção et al., 2021), enabling signaling *via* biochemical and biophysical (spatial organization and mechanical properties) cues (Assunção et al., 2020a). The complex interplay of these properties synergistically enables intricate signaling and the modulation of biological processes (Chiang et al., 2021). It is therefore not surprising that IGFBP7 was shown to act in collaboration with other ECM components and signaling factors, suggesting that IGFBP7’s functional role in angiogenic processes is dependent on the ECM microenvironment and present signaling partners. This notion is supported by the fact that IGFBP7 has multiple binding partners within the ECM, including Col IV (Akaogi et al., 1996a) and heparan sulfate (Sato et al., 1999), is able to interact with cell surface heparan sulfate and various cell receptors. Integrin αvβ3 is especially noteworthy, as it suggests that IGFBP7 may act *via* mechanotransduction (Komiya et al., 2014; Driscoll et al., 2021), rendering it crucial for IGFBP7 to be properly embedded within the ECM. This understanding gives a possible explanation for the highly conflicting results obtained from research utilizing soluble IGFBP7 and suggests that they may not be as physiologically relevant.

Having established that IGFBP7’s bioactivity is microenvironment context-dependent, its contradictory effect on the activity of its various binding partners, such as IGF (Akaogi et al., 1996b; Evdokimova et al., 2012; Akiel et al., 2017; Artico et al., 2021) and VEGF (Hooper et al., 2009; Tamura et al., 2009) could also be explained. Indeed, the presence of synergistically promoting components or competitive ligands (e.g., presence of IGFBPs with higher affinity for IGF), relative abundance of relevant receptors on the cell surface, and cellular identity may be decisive in the role of IGFBP7 in angiogenesis. Moreover, IGFBP7’s ability to directly bind other cytokines and chemokines (Nagakubo et al., 2003) also opens the avenue of IGFBP7 acting as a signaling factor reservoir, where it can bind, sequester and/or present them to cells.

Consequently, IGFBP7 role in angiogenesis will be dependent on the composition of the local microenvironment, which is starkly different during development, physiological tissue healing and remodeling, as compared to the pathological context of the TME, dysregulated wound healing, NAFLD, and failing myocardium. Moreover, its effect may be strongly influenced by temporal, spatial, and concentration-dependent dosing. Nonetheless, current studies suggest that IGFBP7 promotes endothelial cell attachment, luminogenesis, and potentially vessel stabilization and maturation during physiological angiogenesis. Its effects on earlier stages of angiogenesis including endothelial cell proliferation, migration, and initial stages of sprouting, as well as vascular barrier functions, remain to be determined. It also modulates the pro-angiogenic properties of other signaling factors, such as VEGF and IGF, and their downstream signaling, introducing an additional level of complexity.

Besides vascularization, IGFBP7 is evidently involved in other biological processes in tissue development and remodeling. It has a promoting role in progenitor and stem cell commitment and is likely anti-inflammatory and anti-fibrotic. Nonetheless, its role in inflammation, immunomodulation, fibrosis, and cellular senescence is again likely to be context-dependent.

Future studies should thus explore the function of IGFBP7 in (re-)vascularization and tissue remodeling within its inherent microenvironment and compare it to functions of its soluble counterpart. This could involve *in vivo* studies with temporal tissue- or cell-specific depletion or over-expression of IGFBP7, as well as *in vitro* studies involving other ECM components, decellularized tissue-derived or cell culture-derived ECM. Especially *in vitro* studies would allow to introduce changes into the ECM microenvironment and investigate how these changes affect IGFBP7’s bioactivity, as well as explore involved molecular pathways. Studies in microphysiological systems and more complex physiologically relevant co-cultures will further allow to investigate how different cell sources of IGFBP7 and bioactive co-factors affect its biological functions. Advanced studies are thus required to shed more light on the intricate functioning of IGFBP7.

## Figures and Tables

**Table:** 

**AGM**	Angiomodulin
**ALL**	Acute Lymphoblastic Leukaemia
**Akt**	Protein kinase B
**ANG-1**	Angiopoietin-1
**ASC**	Adipocyte-derived stem cells
**α-SMA**	Alpha Smooth Muscle Actin
**BBB**	Blood-Brain-Barrier
**βig-h3**	Transforming Growth Factor-β–induced Protein ig-h3
**BMP**	Bone Morphogenetic Protein
**BMP2**	Bone Morphogenetic Protein 2
**CAF**	Cancer Associated Fibroblast
**CAM**	Chicken Chorioallantoic Membrane
**CCL5**	C-C motif chemokine 5
**CCL21**	C-C motif chemokine 21
**CEC**	Circulating Endothelial Cells
**CLDN5**	Claudin-5
**CNS**	Central Nervous System
**Col I**	Type I Collagen
**Col IV**	Type IV Collagen
**COX-2**	Cyclooxygenase-2
**CXCR-4**	C-X-C chemokine receptor type 4
**CXCL10**	C-X-C motif chemokine 10
**ECM**	Extracellular Matrix
**EFNB2**	Ephrin B2
**EOC**	Epithelial Ovarian Cancer
**ERK**	Extracellular Signal-Regulated Kinase
**ERK1/2**	Extracellular Signal-Regulated Kinase 1/2
**ESC**	Embryonic Stem Cells
**FGF-2**	Fibroblast Growth Factor-2
**FGFR-1**	Fibroblast Growth Factor Receptor 1
**FOXO3a**	Forkhead box O3
**GBM**	Glioblastoma
**GSK-3 beta**	Glycogen Synthase Kinase-3 Beta
**HCC**	Human Hepatocellular Carcinoma
**HGF**	Hepatocyte Growth Factor
**HOMEC**	Human Ovarian Microvascular Endothelial Cells
**HUVEC**	Human Umbilical Vein Endothelial Cells
**IGF**	Insulin-like Growth Factor
**IGFBP**	Insulin-like Growth Factor Binding Protein
**IGFBP-rp1**	Insulin-like Growth Factor Binding Protein-related Protein 1
**IGFBP7**	Insulin-like Growth Factor Binding Protein 7
**IGF-I**	Insulin-like Growth Factor I
**IL-6**	Interluekin-6
**IL-8**	Interluekin-8
**LacZ**	β-galactosidase
**LEC**	Luteal Microvascular Endothelial Cells
**LH**	Luteinizing Hormone
**MAPK**	Mitogen-Activated Protein Kinase
**MFSD2A**	Major Facilitator Superfamily Domain-Containing Protein 2
**MSC**	Mesenchymal Stem Cells
**mTOR**	Serine/threonine-protein kinase mTOR
**NAFLD**	Non-Alcoholic Fatty Liver Disease
**PCOLCE**	Procollagen C Endopeptidase Enhancer 1
**PGE2**	Prostaglandin E2
**PI3K**	Phosphoinositide 3-kinases
**PTH**	Parathyroid Hormone
**TAF**	Tumor-derived Adhesion Factor
**PSF**	Prostacyclin-Stimulating Factor
**SMC**	Smooth Muscle Cells
**SPARC**	Secreted protein acidic and rich in cysteine
**TGF-α**	Transforming Growth Factor alpha
**TGF-β**	Transforming Growth Factor beta
**TGF-β1**	Transforming Growth Factor beta 1
**TGF-β3**	Transforming Growth Factor beta 3
**TAM**	Tissue Associated Macrophages
**TME**	Tumor Microenvironment
**TNF**	Tumor Necrosis Factor
**TNFα**	Tumor Necrosis Factor-α
**VEGF**	Vascular Endothelial Growth Factor
**VEGF-A**	Vascular Endothelial Growth Factor A
**VEGFR-2**	Vascular Endothelial Growth Factor Receptor 2
**VE-Cadherin**	Vascular endothelial cadherin
**4E-BP1**	Eukaryotic translation initiation factor 4E-binding protein 1
